# Targeting Histone H3K9 Methyltransferase G9a as a Potential Therapeutic Strategy for Neuropsychiatric Disorders

**DOI:** 10.1002/med.22119

**Published:** 2025-05-19

**Authors:** Malak Hajar, Tobias Werner, Mihajlo Gajic, Holger Stark, Bassem Sadek

**Affiliations:** ^1^ Department of Pharmacology & Therapeutics, College of Medicine and Health Sciences United Arab Emirates University Al Ain UAE; ^2^ Zayed Bin Sultan Centre for Health Sciences United Arab Emirates University Al Ain UAE; ^3^ Institute of Pharmaceutical and Medicinal Chemistry Heinrich Heine University Düsseldorf, Universitaetsstr. 1 Düsseldorf Germany

**Keywords:** Alzheimer's disease, anxiety, autism spectrum disorders, depression, epigenetics, epilepsy, G9a/EHMT2, histone lysine methyltransferases, inhibitors, neuropsychiatric disorders, Prader‐Willi syndrome, schizophrenia

## Abstract

Neuropsychiatric disorders present a multifaceted challenge, characterized by cognitive, social, and motor impairments with manifold underlying mechanisms. Recent attention has turned to epigenetic mechanisms, particularly histone lysine methyltransferases (HKMTs), such as G9a, in understanding fundamental pathogenesis. This review provides a concise overview of the structural and functional features of G9a and its involvement in neuropsychiatric disorders, including neurodevelopmental disorders (NDDs) like autism spectrum disorders (ASD) and Prader‐Willi syndrome (PWS), schizophrenia (SZ), epilepsy, anxiety, depression, and Alzheimer's disease (AD). Furthermore, it highlights the biochemical mechanisms of G9a‐mediated histone methylations and explores pharmacological interventions targeting G9a for potential therapeutic avenues. This current knowledge underlines G9a's significance as a therapeutic target and sets the stage for future investigations into its role in neuropsychiatric disorders.

## 1 Introduction

Neuropsychiatric disorders represent a complex spectrum of conditions that affect both the nervous system and mental health. Fundamentally, neuropsychiatric disorders, whether neurodevelopmental, neurological or psychiatric, arise from altered functions of the nervous system, leading to cognitive, social, and motor skills impairments that emerge during early development or at later stages of an individual's lifespan and can persist throughout [1]. They are characterized as chronic and mostly incapacitating conditions that, according to a recent WHO report, account for 10% of disease‐related disabilities worldwide, posing not only an immense social, but also economic burden [2].

Due to their multifaceted nature and accompanying comorbidities, the underlying mechanisms remain largely uncharacterized [3, 4]. Recently, epigenetic mechanisms have emerged as key regulators of normal neural functions. The term epigenetics refers to heritable changes in gene expression that do not involve the alteration of the DNA sequence itself, but rather rely on mechanisms like DNA methylation, histone modification, chromatin remodeling and noncoding RNA‐based regulation [5, 6, 7]. The role of epigenetics in neuropsychiatric disorders stems from its regulation of dynamic gene expression patterns necessary for generating the diverse cell types essential for proper brain function [8].

This epigenetic system involves important chromatin remodelers, broadly classified into the groups of epigenetic writers, readers and erasers. Chromatin remodelers attracted great attention as drug discovery targets, such as histone deacetylases (HDACs) or histone lysine methyltransferases (HKMTs). As implied by their nomenclature, HKMTs are responsible for the methylation of lysine residues located on histone tails. Leading to transcriptional activation or repression, these highly expressed enzymes contribute to the regeneration, fate differentiation and fate determination of cells, as well as sustaining DNA integrity and replication [9, 10]. One of the most studied transcriptional repression modifications is histone 3 lysine 9 (H3K9) methylation by G9a methyltransferase. G9a, also referred to as euchromatic histone‐lysine *N*‐methyltransferase 2 (EHMT2), along with its paralog, G9a‐like protein (GLP) or EHMT1, are mainly responsible for the mono‐ and di‐methylation of H3K9 [4, 11, 12, 13]. Histone methyltransferases have been extensively studied as pathophysiological contributors and therapeutic targets in a wide range of conditions, including cancers [14, 15, 16], autoimmune [17], inflammatory [18, 19], and neurodegenerative disorders [20]. Recently, there has been growing interest in exploring the potential involvement of histone methyltransferases, specifically methylation of H3K9 by G9a methyltransferase in neuropsychiatric disorders [21, 22, 23, 24], which manifested as efforts of targeting G9a pharmacologically with small molecule inhibitors [21, 25, 26, 27]. During the revision of this paper, an excellent review article by Bellver‐Sanchis et al. on G9a and its therapeutic potential was published [28].

The rapidly growing interest and expanding body of evidence surrounding the epigenetic components of neuropsychiatric disorders underscore the need for systematization of accumulated knowledge. The current paper briefly aims to provide outlines of structural and functional features of G9a, followed by a concise overview of its roles in the selected neuropsychiatric disorders including autism spectrum disorders, Prader‐Willi syndrome, schizophrenia, epilepsy, anxiety, depression, and Alzheimer's disease, as well as to highlight recent trends in the development of G9a inhibitors [15, 29, 30, 31, 32, 33]. Numerous other aspects of G9a cannot be considered due to place restrictions.

## 2 H3K9 Methyltransferase G9a: Structural and Functional Features

The G9a gene, also known as EHMT2, is located on the long arm of chromosome 6 (6p21.33) [34]. The canonical isoform of G9a or isoform A, comprises approximately 1210 amino acid residues in humans, while the shorter splice variant Isoform B on the other hand is made up of 1176 amino acid residues [12]. Alternative splicing not only impacts enzyme lengths but also significantly influences their functional properties [35]. For instance, exon 10 inclusion in G9a long isoform is necessary for neuron differentiation and can affect its own pre‐mRNA splicing, creating a positive feedback loop [36].

**Figure 1:**
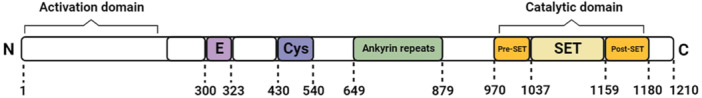
Schematic representation of the structural domains of human G9a. [Color figure can be viewed at wileyonlinelibrary.com]

The domain architecture of G9a includes an N‐terminal activation domain located upstream, followed by a glutamate‐rich domain (E), a cysteine‐rich domain (Cys), ankyrin repeat units, and a C‐terminal catalytic Su(var)3‐9, Emhancer‐of‐zeste and Thithorax (SET) domain, as shown in Figure 1. The N‐terminus of G9a contains a nuclear localization signal (NLS) and is therefore responsible for its nuclear localization [37]. G9a and its paralog, G9a‐like protein (GLP), share a remarkable 45% sequence identity and 70% sequence similarity, differing mainly in their N‐terminal region and in the polyglutamate (E) domain, where GLP features a distinct repetitive pattern of aspartic and glutamic acids [38]. The ankyrin repeat domain functions as a reader module for mono‐ and di‐methyl lysine binding, with G9a generally exhibiting higher affinity for mono‐methylated H3K9 [39]. Within the catalytic region, the SET domain of G9a and GLP shares over 80% sequence identity and is flanked by pre‐SET and post‐SET domains that collectively facilitate cofactor binding, substrate recognition, and protein stability [40]. Mutations in the SET domain disrupt di‐ and tri‐methylation of histone H3K9 [41].

Several studies have shown that aromatic residues in the post‐SET domain enhance methyltransferase activity by stabilizing the interaction between the adenine portion of S‐adenosylmethionine (SAM) and the enzyme, facilitating the exposure of the methyl group for transfer [42]. Trievel et al. proposed a mechanism where the catalytic residue Tyr‐287 connects the cofactor and lysine binding sites, playing a crucial role in methyl transfer [43]. Additionally, the SET domain maintains its enzymatic activity through its interaction with the post‐SET domain, stabilized by Tyr372 and Phe374 [44]. The SET domain also relies on four zinc finger regions for proper folding and function. Three Zn²⁺ ions are coordinated by nine cysteine residues, while a fourth Zn²⁺ ion, located near the SAM‐binding site, is bound by four cysteine residues. The cysteine‐rich domain interacts with these structural zinc ions, which are essential for the catalytic activity of G9a [42]. G9a and its paralog GLP form homo‐ and heterodimers via their SET domains and interact with zinc finger proteins such as ZNF644 and WIZ. This SET‐ZF complex plays a key role in recognizing and binding specific DNA sequences to regulate H3K9 methylation [45].

**Figure 2:**
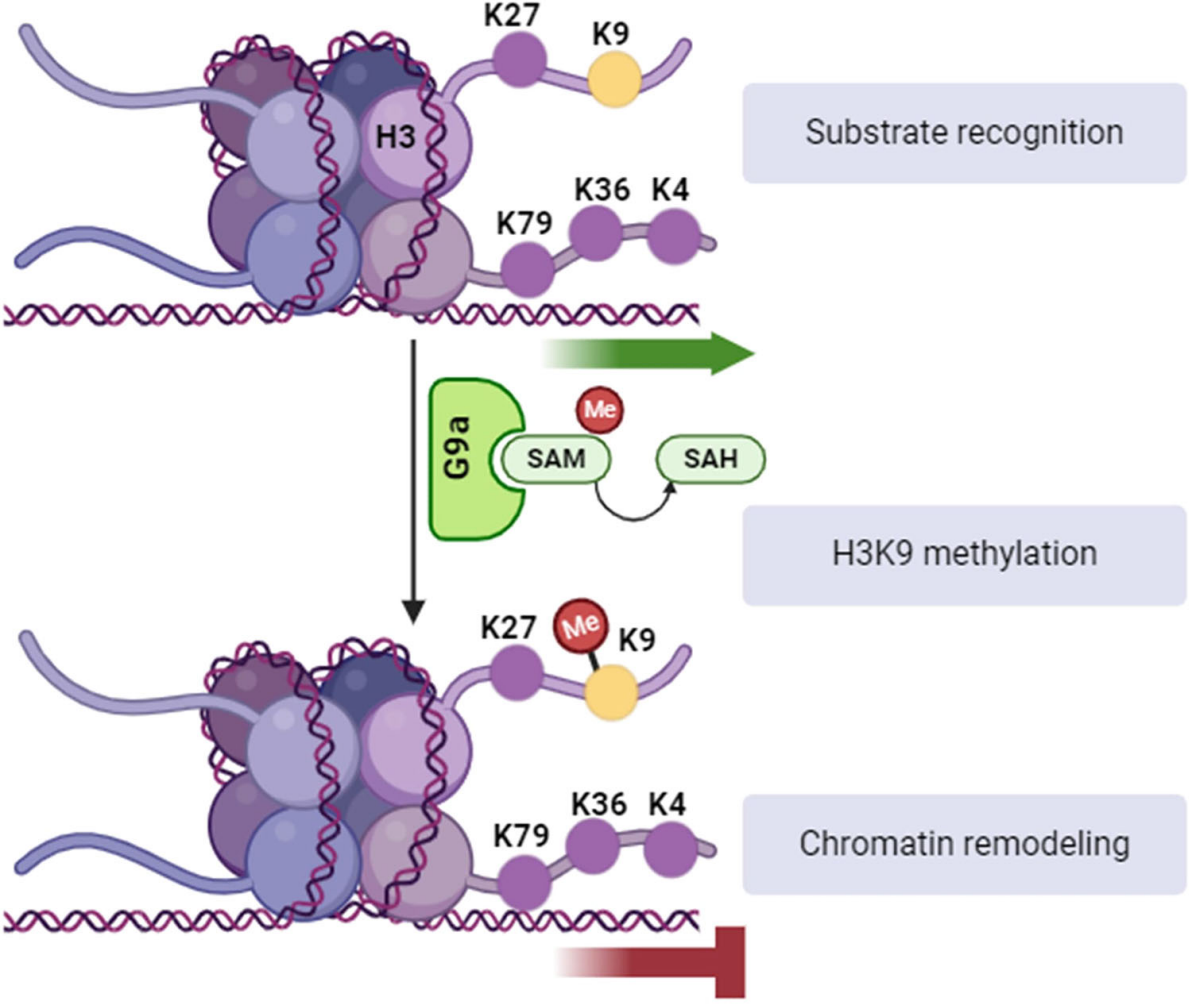
Schematic representation of the mechanism of action of G9a on chromatin. [Color figure can be viewed at wileyonlinelibrary.com]

Biochemical studies have extensively examined the mechanism of G9a methylation [46, 47], breaking it down into three key steps, illustrated in Figure 2. The SET domain of G9a enzyme recognizes the lysine on position 9 (K9) on histone H3 tail and catalyses the donation of SAM methyl group to ɛ‐amino group of K9 resulting in S‐adenosyl homocysteine (SAH) [48, 49]. H3K9 methylation is commonly associated with the formation of a repressive chromatin state via recruiting other related proteins such as heterochromatin protein 1 (HP1) which in turn can promote chromatin compaction and gene silencing [50]. Y1067 in the catalytic centre of G9a determines whether mono‐, di‐ or, after an increased incubation period, trimethylation takes place [51, 52]. In vitro experiments with truncated histone H3 tails showed that methylation reaction by G9a requires at least seven amino acids (TARKSTG) [10].

## 3 H3K9 Methyltransferase G9a Role in Neuropsychiatric Disorders

**Figure 3:**
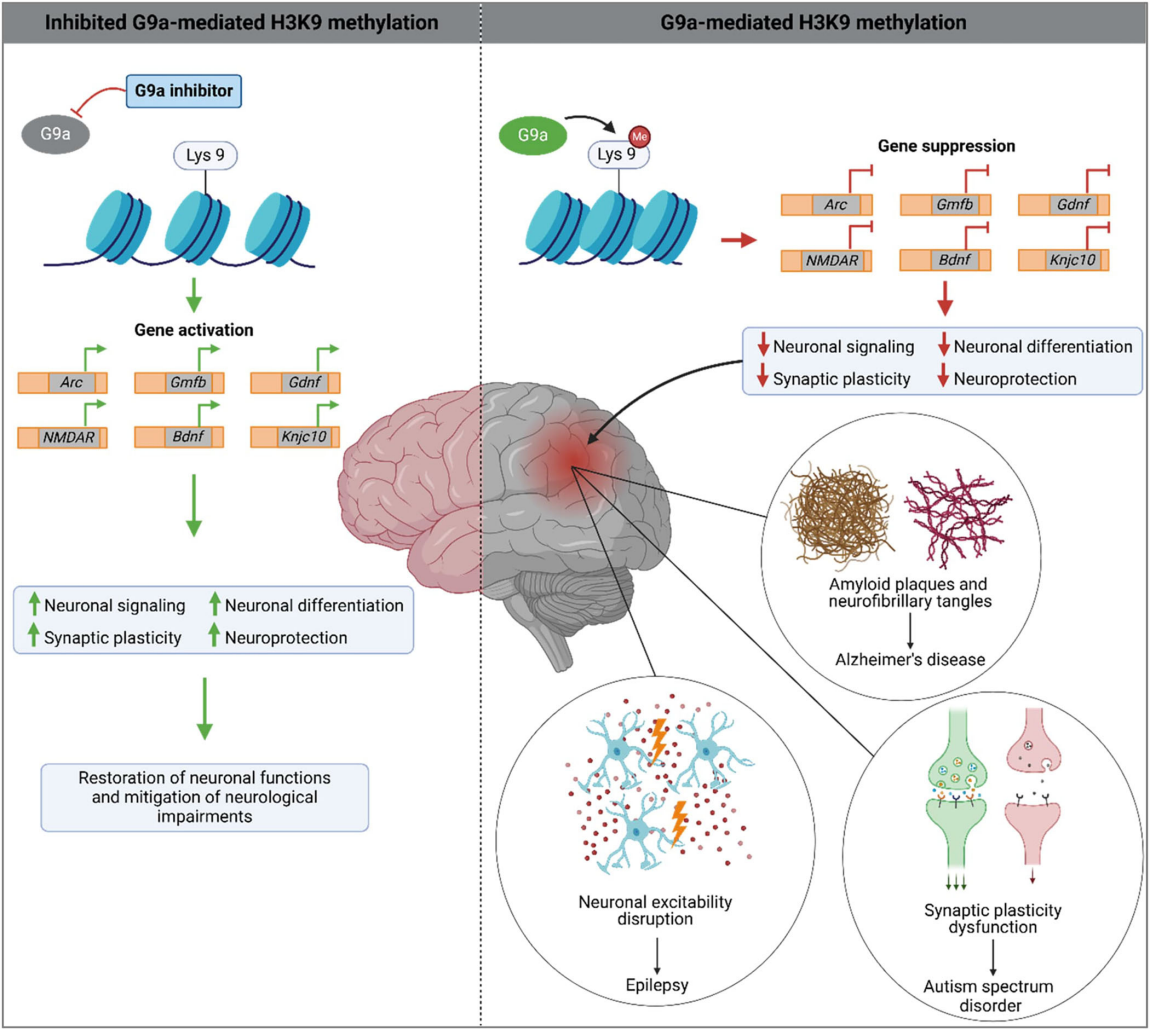
Schematic representation of G9a's role in selected disorders and the effects of its inhibition on neuronal function. *Arc*: Activity‐regulated cytoskeleton‐associated protein; *Bdnf*: Brain‐derived neurotrophic factor; *Gmfb*: Glia maturation factor beta; *Gdnf*: Glial cell line‐derived neurotrophic factor; Kcnj10: Potassium inwardly‐rectifying channel subfamily J member 10; *NMDAR*: N‐Methyl‐d‐Aspartate Receptor. [Color figure can be viewed at wileyonlinelibrary.com]

Dysregulation of G9a‐mediated H3K9 methylation has been implicated in various neurodevelopmental and neuropsychiatric disorders, contributing to gene suppression and neuronal dysfunction. Figure 3 provides an overview of disorders where G9a‐mediated gene suppression has been specifically reported and illustrates the effects of its inhibition on neuronal function.

### 3.1 Autism Spectrum Disorder

Autism spectrum disorders (ASDs) are a combination of complex neurodevelopmental disorders characterized by repetitive stereotyped behaviors and impaired social communication [53]. On a global scale, the prevalence of ASDs has witnessed a rise of 20‐ to 30‐fold in the last 40 years with males being approximately four times more affected than females [54, 55]. A variety of genetic and environmental factors have been attributed to the development of ASDs, and interestingly, histone‐modifying enzymes that control histone methylation/demethylation are being reported as key risk factors in ASDs [56, 57]. G9a‐mediated H3K9 methylation was shown to suppress the expression of genes associated with neural signaling such as Arc and NMDA receptor genes in Shank‐deficient mice. This leads to impaired synaptic plasticity and cognitive deficits [58].

Schaefer et al. found that deficiency in GLP/G9a in the postnatal mouse brain is sufficient to cause complex behavioral changes such as reduced locomotion and exploration [59]. Treatment with highly selective LSD1 inhibitor GSK‐LSD1 [60] and G9a inhibitor UNC0642 [58], resulted in the restoration of a significant number of genes highlighting the therapeutic potential of targeting this enzyme. Additionally, Kleefstra syndrome is a rare genetic disorder characterized by autistic‐like features [61]. Iacono et al. performed behavioral phenotyping of mice with different genetic backgrounds with the goal to investigate the cognitive dysfunctions associated with Kleefstra syndrome. Obtained results revealed that haploinsufficiency of GLP counterintuitively resulted in elevated H3K9 methylation and diminished protocadherin expression in the brain, aligning with cognitive impairments. Interestingly, haploinsufficiency of paralogue G9a did not produce similar cognitive deficits, suggesting evolutionary divergence in functional roles [62]. ASDs are also comorbid with other disorders such as epilepsy, Rett syndrome and Fragile X syndrome [63]. In the latter syndrome, silenced genes were associated not only with elevated levels of histone H3 di‐methylation at lysine 9 (H3K9me2), but also with H3K9Me3, H3K27Me3, and H4K20Me3 [64]. Interestingly, epigenetic regulation by G9a (Ehmt2) also influences TRPA1 and its closely related TRPV1 channels, whose upregulation contributes to chronic inflammatory pain and may promote metabolic dysfunction frequently observed in ASDs, suggesting that targeting these channels could be a novel therapeutic strategy to mitigate both pain and metabolic comorbidities in neurodevelopmental disorders [65, 66].

### 3.2 Prader‐Willi Syndrome

Prader‐Willi syndrome (PWS) is an orphan neurodevelopmental disorder with an estimated prevalence of 1 in 15,000–30,000 [67]. It manifests through hypotonia, sucking difficulties in newborns, excessive eating behavior in later infancy and, as a result, morbid obesity as the earliest clinical symptoms, that are accompanied by delayed motor development, behavioral disorders, mental retardation, hypogonadism, incomplete pubertal development, sleep disorders and short stature [68]. PWS is caused by a lack of paternally expressed genes in the 15q11‐q13 chromosomal region. However, the genes remain on the maternal chromosome but are epigenetically suppressed [67]. Given its unique epigenetic mechanism, PWS presents a promising model for exploring epigenetic therapy, as reactivating these genes could offer therapeutic benefits. It has been shown that the G9a inhibitors UNC0638 and UNC0642 are able to restore the expression of PWS‐associated genes of the maternally inherited chromosome by selectively reducing the dimethylation of histone H3 without altering DNA methylation. Kim et al. treated newborn PWS mice with UNC0642 leading to improved growth and survival as a proof of principle for a PWS therapy based on epigenetics [69]. Building on these findings, a new G9a/EHMT2 inhibitor, MS152, with enhanced brain penetration and oral bioavailability, effectively reactivates maternal PWS genes and alleviates perinatal lethality and poor growth [70]. A recent study on potent G9a inhibitor A‐366 demonstrated its high affinity at histamine H_3_ receptor (H_3_R) as G‐protein coupled receptor indicating a potentially beneficial therapeutic effect of simultaneous targeting of G9a and H_3_R [71], given that the treatment with known H_3_R inverse agonist Pitolisant led to cognitive improvements in patients suffering from PWS [72].

### 3.3 Schizophrenia

Schizophrenia (SZ) is a chronic and complex neuropsychiatric disorder that affects ~1% of the global population. It is characterized by clusters of positive, also known as psychotic symptoms, encompassing delusions, hallucinations and cognitive impairment, as well as negative symptoms such as avolition (lack of motivation), anhedonia (loss of ability to feel pleasure) and social withdrawal [73, 74]. Although no sex differences were reported in lifetime prevalence, schizophrenia is more common in men, with a ratio of approximately 1.4:1 [75]. Proneness to SZ involves physical, psychological, and environmental elements. Yet, increasing evidence indicated a correlation between SZ and epigenetic abnormalities in genes associated with neurotransmission, neurodevelopment, and immune function [76]. Apart from G9a and GLP, that are responsible for the majority of H3K9me2 modifications across the genome, SETDB1 is another euchromatic histone methyltransferase (HMT) that has been linked to SZ with the role to specifically perform di‐ and tri‐methylation of H3K9 [4]. Chase et al. assessed H3K9me2 levels in both brain and blood tissues. Their findings revealed elevated G9a levels in lymphocyte samples from schizophrenic individuals, along with increased G9a and SETDB1 expression in both lymphocytes and postmortem parietal cortex. These results were replicated in a following genome‐wide sequencing study on the frontal cortex [77]. Additionally, mRNA levels of RE1‐silencing transcription factor (REST) responsible for coordinating heterochromatin assembly alongside other repressor proteins were relatively diminished in SZ individuals [78]. Concomitant upregulation of HMTs accompanied by downregulation of REST protein expression indicates a restrictive chromatin environment in SZ individuals, which might play a prominent role in the pathology of SZ.

### 3.4 Epilepsy

Epilepsy belongs to neuropsychiatric disorders characterized by unprovoked, spontaneous and recurrent seizures. This chronic condition affects more than 50 million patients worldwide. Seizures are a result of unbalanced excitation and inhibition of brain neurons [79, 80]. In addition to sex differences in seizure expression, reproductive/sex hormones can also influence seizure susceptibility. Besides their main role in the tissue of reproductive organs, sex hormones are critical for normal brain functions through affecting and regulating neuronal excitability and survival. Thus not surprisingly, clinical and experimental studies show that seizures reflect changes in sex hormone levels; in some women with epilepsy, seizure exacerbation can be related to periodical hormonal fluctuations during the ovarian cycles, a condition called catamenial epilepsy [81]. The role of epigenetic mechanisms, including DNA methylation and posttranslational histone modifications, in epileptogenesis has been highlighted in the literature [82, 83, 84, 85]. Significant alterations in lysine acetylation, marked by increased expression of histone deacetylase 2 (HDAC2), have been observed in individuals with acquired temporal lobe epilepsy [86]. Also, other HDACs activity on glutamate receptor (GluR2) was proven related to neuronal hyperexcitability [87]. Therefore, many antiepileptic drugs, particularly valproic acid (VPA), act as HDACs inhibitors [84]. Additionally, VPA modulates promoter DNA methylation in hippocampus, thereby altering gene expression and cell proliferation [88]. In a study by Zhang et al., G9a was found to influence the expression of *Kcnj10* channel, an ATP‐sensitive inward rectifier potassium channel that regulates neuronal excitability, action potential dynamics, and neuronal synchronization [89]. Histone lysine methylation has not been thoroughly examined in the context of epilepsy. Nevertheless, a recent study focusing on polycomb repressive complexes 1 and 2 (PRC1 and PRC2), responsible for regulating the trimethylation of histone H3 at lysine 27 (H3K27me3), a recognized posttranslational histone modification linked to transcriptional repression, H3K27me3 was found to influence the expression of matrix metalloproteinase‐9 during seizures [90]. Another recent study by Zhang et al., reported G9a‐mediated increases in H3K9me2 levels, silences genes such as Slo2.2 (a potassium sodium‐activated channel) and thereby contribute to heightened neuronal excitability seen in conditions like opioid‐induced hyperalgesia [91].

### 3.5 Anxiety and Depression

Anxiety disorders (ADs) and depression are among the most common and highly comorbid neuropsychiatric disorders. Their onset typically peaks during adolescence and early adulthood, with females at significantly higher risk. Women have twice the lifetime prevalence of depression and most ADs compared to men [92, 93]. Acute severely traumatic events and chronic stress can significantly alter gene expression in the brain, resulting in depressive‐like behaviors [94]. Decreased levels of G9a and associated H3K9 methylation have been observed in the nucleus accumbens (NAc) of stress‐susceptible mice, which correlates with altered expressions of genes critical for mood regulation, including glial cell line‐derived neurotrophic factor (*Gdnf*) and brain‐derived neurotrophic factor *(Bdnf*) [95]. Hence, recent studies have focused on epigenetic enzymes as a potential therapy target. For instance, the use of HDAC inhibitors not only enhanced the activity of fluoxetine, a selective serotonin reuptake inhibitor (SSRI), but also had an effect on SSRI non‐responders [96]. In mice previously exposed to stress, a study by Xu et al. concluded that chronic treatment with the antidepressant tranylcypromine inhibited the methylation of lysine 9 on histone 3 (H3K9me2) by G9a [97]. In terms of behavioral influence, the inhibition of G9a/GLP by potent compounds like UNC0642 and A‐366 have decreased anxiety‐like behaviors and increased social ability in adult mice, along with a reduction in H3K9 methylation levels in the brain. However, both compounds when administered to pregnant mice, the offspring displayed increased anxiety levels and reduced social interaction [21]. Recent studies on an animal model with rats showing depressive‐like behaviors revealed increased protein levels of H3K9me2 in the hippocampus and medial prefrontal cortex and interestingly, decreased protein levels if UNC0642 was administered to the rats [98].

### 3.6 Alzheimer's Disease

Alzheimer's disease (AD) is a neuropsychiatric disorder characterized by slow, irreversible and progressive deterioration of neurons, resulting in cognitive and behavioral impairments [99]. It is the most common form of dementia estimated to affect more than 400 million people worldwide [100]. A multifaceted nature of complex AD etiopathology is represented through converging network of genetic, epigenetic, biological and environmental factors [101]. A hallmark of AD are extracellular plaques of accumulated amyloid beta (Aβ) filaments originating from proteolytically cleaved amyloid precursor protein by β‐secretase [99]. Apart from Aβ plaques, impaired protein metabolism leads to formation of intracellular neurofibrillary tangles, which collectively are in turn accompanied by neuroinflammation and AD characteristic synaptic failure [99, 101]. By exploring the epigenetic impact on AD, a recent study observed increased levels of H3K9me2, G9a, and GLP in the mouse model of late‐stage familial AD, as well as in post‐mortem tissues from human AD patients. A treatment of mice with specific G9a/GLP inhibitors not only reversed hypermethylation of histone protein but also rescued synaptic signaling and cognitive functions [102]. Furthermore, beneficial effects of G9a/GLP inhibition were mediated by *Bdnf* through amelioration of Aβ‐induced deficits in late‐long‐term potentiation and synaptic plasticity [103]. Research by Bellver‐Sanchis et al. recently indicated to a novel G9a‐mediated mechanism of neuroprotection via regulation of glia maturation factor β (*Gmfb*). A transcriptional profiling after G9a inhibition treatment led to increased *Gmfb* levels, which was followed by improvements in cognition and neuronal plasticity, while reducing neuroinflammation. Observed neuroprotective effects were abolished by the pharmacological inhibition of *Gmfb*. Moreover, alongside its role in regulating gene expression of *Gmfb* and thus controlling its levels, G9a also modulates *Gmfb* activity through direct methylation of its lysine residues [20]. In cells treated with hydrogen peroxide to cause oxidative stress, inhibition of G9a led to increased cell survival and upregulation of antioxidant enzymes. This observed neuroprotective effect could be due to downregulation of miR‐128 and thus to PPARγ signaling pathway modulation [20]. Recent research suggested a new mechanism of AD pathogenesis based on aberrant G9a activity leading to noncanonical translational regulation of proteins that delineates proteopathologic aspect of AD in hippocampal region. Inhibition of G9a in mice model of AD fully restored cognitive and noncognitive behavioral functions [104].

## 4 H3K9 Methyltransferase G9a Inhibitors

The obvious contribution of G9a in a wide range of neuropsychiatric disorders, in addition to its well‐defined role in cancer pathogenesis [105], prompted G9a as an attractive pharmacological target. After almost two decades since the report of first G9a inhibitor, BIX‐01294 [106], the scientific endeavor to find novel, efficacious and safe G9a inhibitors covered a wide range of structural scaffolds, primarily dominated by quinazolines and quinolines, but also spanning over benzodiazepines, amino‐indoles and 2,4‐diamino‐6‐methylpyrimidines among others [107]. Based on the mechanism of action, G9a inhibitors could be broadly classified in two main groups, namely substrate (protein) competitive and SAM cofactor competitive inhibitors [108] followed by a relatively limited number of compounds that achieve G9a inhibition through ejection of Zn^2+^ from Zn‐finger motifs [109]

Despite great efforts to utilize the promising therapeutic profile of G9a inhibitors, multiple clinical trials have been discontinued due to poor pharmacokinetics and safety concerns. As a result, none of the known G9a inhibitors is currently undergoing clinical trials [108, 110], leaving not only a long journey for the first drug to reach market, but also further necessitating G9a‐focused drug development programs.

### 4.1 Substrate Competitive Inhibitors

BIX‐01294 is among the first G9a inhibitors identified from a large library of small molecules by Kubicek et al. [106]. This diazepinquinazolin‐amine derivative (Table 1) selectively inhibits G9a and at higher concentrations also impairs the closely related GLP, via specifically competing with the amino acids located on the N‐terminal side of the histone lysine residue [38]. Effects of BIX‐01294 on apoptosis, autophagy and cell proliferation have been reported especially in cancer cells including T lymphoblastic leukemia cells [127], bladder cancer [128], nasopharyngeal cancer [129], breast and colon cancer cells [130]. BIX‐01294 has been shown to be active against neurodegenerative and neurodevelopmental disorders as well. In a clinical trial investigating H3K9me2 levels and mRNA expression in peripheral blood mononuclear cells and in the mice cortex, BIX‐01294 demonstrated an upregulating effect on schizophrenia‐associated genes including IL‐6, GAD67, NANOG, and KLF4. Additionally, BIX‐01294 counteracted the downregulation of *Bdnf* observed in the striatum of the brain [77, 111]. Although cellular toxicity prevented BIX‐01294 from advancing into clinical trials, its molecular structure guided lead optimization, leading to the discovery of more selective and potent G9a inhibitors known as the UNC series [106]. Liu et al. hypothesized that lysine binds to a tunnel within histone 3. To enhance G9a specificity and, consequently, potency, they strategically employed linkers to extend BIX‐01294 into this lysine tunnel. This approach resulted in the discovery of UNC‐0224 and UNC‐0321, exhibiting IC_50_ values of 15 and 6 nM, respectively (Table 1). Both compounds lacked drug‐like properties and exhibited cytotoxicity issues [121, 122]. Hence, Liu et al. proceeded with optimization by modifying three different positions of the quinazoline scaffold. The propoxy pyrrolidine side chain of UNC‐0638 at position 7 made it not only equipotent against G9a and GLP, but it also had lower cytotoxicity and wider therapeutic window [123]. In fact the propoxy pyrrolidine of UNC‐0638 was hybridized with EZH2 pharmacophore in a recent study by Shi et al., resulting in a G9a/EZH2 dual inhibitor with an IC50 of 2.9 nM on G9a [131]. In a recent study, the reduction of H3K9me2 levels by UNC‐0638 was linked to an enhanced cortical expansion [124]. Lastly, UNC‐0642 was designed by substituting the metabolically labile cyclohexane with a 4,4‐difluoropiperidine. This resulted in a tenfold increase in plasma concentrations enhancing its in vivo potency [132]. The excellent CNS penetration of UNC‐0642 made it a suitable candidate to test its effect on a m + /p∆S − U mouse model of PWS. It was shown that UNC‐0642, not only improved the lifespan and weight gain of m + /p∆S − U pups but also produced long‐lasting activation of PWS‐associated genes such as SNORD116. Importantly, the treatment was apparently well tolerated, exhibiting no notable acute toxicity [69]. In another study UNC‐0642 both prevented and restored ethanol‐induced loss of choline acetyltransferase immunoreactive basal forebrain cholinergic neurons, perceived as suppression of the cholinergic neuron phenotype [133], Further optimizations of UNC members yielded MS1262, a novel G9a/GLP inhibitor containing 2‐morpholino‐substituted quinoline core, with IC_50_ values for G9a and GLP of 19 ± 14 and 6 ± 1 nM, respectively. MS1262 exhibited not only 23‐fold higher potency than UNC‐0642, but also showed good brain penetration in an in vivo mouse study and managed to completely revert cognitive and noncognitive impairments in an AD mouse model through direct G9a inhibition [104].

**Table 1:** Overview of compounds targeting G9a, their structures, IC_50_ values, effects on neuropsychiatric disorders, and tested models.

Compound	Structure	IC_50_ (nM) on G9a	Effects on neuropsychiatric disorders	Tested model(s)	Reference(s)
A‐366	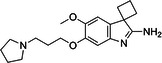	3.3	Reduction of anxiety‐like behavior	C57BL/6 mice	[21, 25]
BIX‐01294	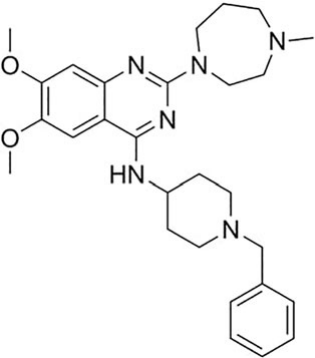	1700	Upregulation of schizophrenia‐associated genes; Reversal of *Bdnf* downregulation in the brain striatum	Mice model; Human schizophrenic participants	[21, 106, 111]
BIX‐01338	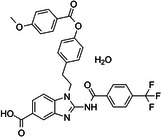	4700	Selectivity limitations		[112]
BRD4770	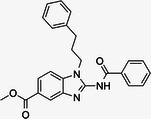	5000	Effects on neuropsychiatric disorders have not been investigated yet		[113]
BRD9536	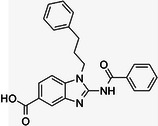	6300	in vitro inactivity		[112]
Chaetocin	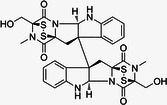	2500	Reduction of neuronal differentiation; Increase in neuronal proliferation.	Adult hippocampal progenitors	[114, 115]
DCG066	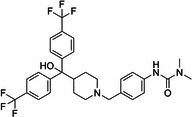	1700	Effects on neuropsychiatric disorders have not been investigated yet		[116]
EML741	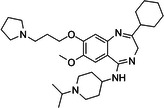	13.8 and 3.6*	Effects on neuropsychiatric disorders have not been investigated yet		[117]
MS1262	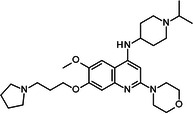	19	Reversion of cognitive and noncocnitive AD impairments	Mice model	[104]
MS8709	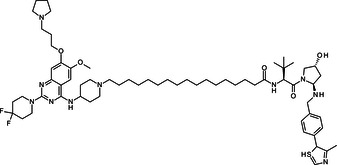	N/A**	Effects on neuropsychiatric disorders have not been investigated yet		[118]
Raltitrexed	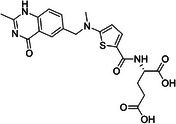	120	Improvement in locomotive functions; Reduction of Aß aggregates	Worm model	[119]
RK‐701	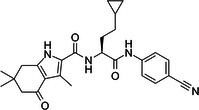	27	Effects on neuropsychiatric disorders have not been investigated yet		[120]
UNC0224	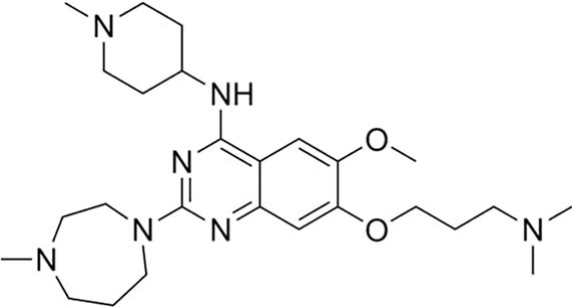	15	Cytotoxicity limitations		[121]
UNC0321	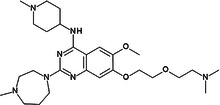	6	Cytotoxicity limitations		[122]
UNC0638	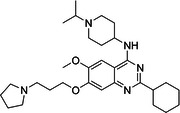	< 15	Promoting cortical expansion; Promoting neural progenitor proliferation	Human cerebral organoids	[123, 124]
UNC0642	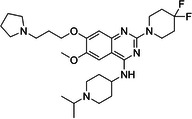	< 2.5	Reversal of autism‐like social deficits; Restoration of NMDA receptors synaptic function	Shank3‐deficient mice	[58, 125]
27	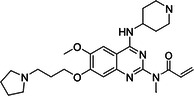	8.5	Effects on neuropsychiatric disorders have not been investigated yet		[126]

* EML741 showed mixed mechanism by acting as competitive substrate inhibitor and noncompetitive SAM inhibitor.

** MS8709 mediates its effect by G9a degradation.

Feng et al. recently reported structure‐based design of covalent G9a inhibitors on the basis of quinazoline and quinoline scaffold. By utilizing various electrophilic warheads targeting cysteine residues (Cys1098 and Cys1186) within the substrate binding domain uniquely present in G9a and GLP, but not other protein lysine methyltransferases, a set of selective and highly potent covalent inhibitors was obtained. The most promising among them, compound 27, showed single digit nanomolar IC_50_ values for G9a and GLP (8.5 and 5.5 nM, respectively), while displaying antiproliferative and antitumor properties [126].

Inspired by UNC‐0638 and devoted to overcoming challenges associated with the quinazoline scaffold, while maintaining potency, a series of G9a inhibitors based on 2‐alkyl‐5‐amino‐ and 2‐aryl‐5‐amino‐substituted 3H‐benzo[e][1,4]‐diazepine scaffold was developed. EML741 is shown to have the highest potency among the series as well as improved selectivity over other methyltransferases, improved permeability in PAMPA and BBB‐PAMPA assays, and low cytotoxicity, all while maintaining the high in vitro and cellular potency of UNC series. Furthermore, EML741 was demonstrated to have mixed mechanism of action by acting as competitive substrate (*K*_i_ = 13.8 ± 1.7 nM) and noncompetitive SAM (*K*_i_ = 3.6 ± 0.6 nM) inhibitor [117]

To confirm that the observed effects of UNC G9a inhibitors are due to G9a and/or GLP inhibition rather than off‐target activity, another class was optimized by substituting its scaffold with a propyl‐pyrrolidine subunit to occupy the enzyme lysine channel (Table 1). One of the most potent non‐quinazoline G9a inhibitors was A‐366 which had been identified by AbbVie, USA research group. Optimized by incorporation of a propyl‐pyrrolidine subunit to occupy the enzyme lysine channel, A‐366 exhibited a remarkable improvement with an IC_50_ as low as 3.3 nM [25]. Several studies confirmed the role of A‐366 in inhibiting growth and differentiation in multiple cancers such as leukemia [134], breast cancer [135], and pancreatic adenocarcinoma [113]. In a study by Wang et al., a series of behavioral tests showed a reduction in anxiety‐like behaviors in adult mice in addition to the decreased brain H3K9me2 levels [21]. In another study of the effect on G9a inhibition of Parkinson's disease (PD), synaptic damage and motor impairment of an α‐synuclein preformed fibrils (PFF)‐induced PD mouse model, was reversed by the A‐366 treatment [136]. This confirms the potential role of G9a and GLP methyltransferases in neuropsychiatric disorders and justifies the targeting of this enzyme in future drug design and development.

Initiative to optimize a lead compound obtained from high‐throughput screening resulted in identification of compound RK‐701, a novel and potent G9a inhibitor (IC_50_ = 27 nM), that demonstrated beneficial effects of G9a inhibition on MOLT‐4 lymphoma cell line, as well as mouse model of carcinogen‐induced hepatocellular carcinoma. Remarkably, RK‐701 is structurally distinct from other known G9a inhibitors, featuring a unique peptidomimetic structure. Unsurprisingly, based on demonstrated potency, favorable pharmacokinetic profile, as well as unmatched structural scaffold among G9a inhibitors, RK‐701 will be a novel drug candidate for G9a‐related diseases [120].

In another study, a systematic 3D quantitative structure–activity relationship (QSAR)‐based high throughput screening on a database of 400,000 compounds was performed with the goal of identifying novel leads for the development of G9a/GLP inhibitors. Conducted analysis highlighted raltitrexed, an anticancer drug, as a final virtual lead competing with substrate binding [119]. Based on the obtained 3D‐QSAR data, raltitrexed was tested in vitro for G9a inhibitory properties exhibiting IC_50_ value of 120 nM. In addition, quantification of H3K9 methylation at different doses in AD transgenic *C. elegans* CL2006 model confirmed direct targeting of G9a by raltitrexed. Using the same Alzheimer's disease transgenic *C. elegans* worms, dose‐dependent improvements in locomotive functions of worms after raltitrexed treatment, accompanied by the reduction of Aβ aggregates by 47%, were demonstrated [119].

### 4.2 Cofactor Competitive Inhibitors

G9a‐catalyzed methylation reaction requires the cofactor SAM as a methyl group donor. Cofactor competitive inhibitors show affinity to SAM binding site on G9a rather than to substrate binding site to compete with SAM preventing substrate methylation due to deficiency of methyl group donor availability. As a consequence of high homology of SAM binding site among methyltransferases, SAM‐competitive G9a inhibitors generally suffer from the lack of selectivity. Chaetocin is a natural product isolated from fungus that acts as SAM‐competitive histone methyltransferase inhibitor with IC_50_ value of 2.5 μM for G9a [137]. BIX‐01338 and BRD9536 displayed problems regarding selectivity, activity or toxicity while BRD4770 provides a more promising pharmacological profile for future developments [78, 96].

### 4.3 Zn^2+^ Ion Ejectors

An alternative approach to inhibiting G9a/GLP aims to exploit their dependence on Zn^2+^ ions for proper folding and enzymatic activity. To date, a relatively limited number of zinc ejectors have been assessed for their G9a/GLP inhibitory properties. Nevertheless, several representatives, including the clinically used ebselen, disulfiram and cisplatin, have shown inhibition of the enzymes at low micromolar and submicromolar concentrations [109]. Although less potent and selective in comparison to substrate and cofactor competitive inhibitors, an important asset of zinc ejectors lies in the well‐established clinical application of some of their representatives, which could serve as inspiration and guidance for further drug development efforts.

### 4.4 Miscellaneous G9a/GLP Inhibitors

A group of miscellaneous G9a/GLP inhibitors consists mostly of newer compounds that, according to current knowledge, do not fit into any mechanism‐based classifications. Their mechanism of G9a/GLP inhibition is either unique or yet to be elucidated.

Aiming to address noncatalytic oncologic functions of overly expressed G9a/GLP enzymes, which were mostly unaffected by conventional inhibitors, Valez et al. developed the first‐in‐class G9a/GLP proteolysis targeting chimera (PROTAC) degrader. The compound MS8709 was obtained by conjugating the parent G9a/GLP inhibitor UNC‐0642 with VHL‐1, as a classical ligand of the VHL E3 ligase, via alkyl linkers. The ability of MS8709 to induce G9a/GLP degradation through recruitment of E3 ligase was demonstrated in several cancer cell lines. Furthermore, the compound exhibited favorable pharmacokinetic properties in a mouse model [118].

A relatively new G9a inhibitor DCG066, that was uncovered by virtual screening, suppressed the growth of leukemia cells through its effects on G9a [118]. A recent study reported positive effects of DCG066 treatment, which inhibited proliferation of multiple myeloma and induced ferroptosis through Nrf2/HO‐1 pathway [136]. Despite observed effects on cancer cell lines, the mechanism of G9a inhibition by DCG066 is still undiscovered. Additionally, a recent study highlighted that the inhibition of G9a not only suppresses the growth of various cancer types but also induces apoptosis and restores normal gene expression patterns, further emphasizing the therapeutic potential in addressing aberrant G9a signaling in cancers [138].

## 5 Conclusions and Future Perspectives

The growing body of research on epigenetic mechanisms, particularly the function of HKMTs such as G9a, has greatly enhanced our understanding of disease pathogenesis across multiple conditions. G9a's critical role in gene expression modulation, especially in maintaining proper neuronal function, positions it as a compelling target for therapeutic intervention. The field of G9a inhibition continues to evolve rapidly, attracting significant attention from drug developers focused on both neurodegenerative disorders and cancer. At the time of revising this article, new compounds targeting G9a have been reported, highlighting the sustained momentum in exploiting its therapeutic potential [139]. Nevertheless, factors such as limited membrane permeability, cytotoxicity, and off‐target interactions with GLP have constrained in vivo efficacy, preventing any G9a inhibitor from advancing into clinical trials. Looking ahead, there are several promising avenues for future research, such as combination therapy. Pairing G9a inhibitors with other epigenetic modulators (e.g., HDAC or DNMT inhibitors) may enhance beneficial gene expression changes, while combining them with immunotherapies could help overcome tumor immune evasion. In the context of neuropsychiatric disorders, co‐administration with histamine H_3_ receptor antagonists or neuroprotective agents may address both epigenetic dysregulation and neurotransmitter imbalances, offering a more comprehensive therapeutic strategy. Additionally, the identification of disease‐specific biomarkers will be central to advancing personalized medicine in G9a research. Possible biomarkers include G9a protein abundance, histone methylation status (e.g., H3K9 dimethylation), and gene expression profiles that reflect G9a‐related pathways. Circulating indicators such as cell‐free DNA or exosomes may also capture G9a‐driven epigenetic changes. Such biomarkers can guide patient stratification and enable more tailored treatment. Ultimately, through strategic collaboration among academia, industry, and regulatory bodies, the development of G9a inhibitors represents a significant opportunity in therapeutic advancement, with broad applications across multiple disease states. This coordinated effort might overcome current limitations and realize the full therapeutic potential of G9a inhibition.

## Author Contributions

B.S. and M.H. were responsible for the study concept, design, and acquisition and analysis of data. M.H., T.W., M.G. and B.S drafted the manuscript. H.S. provided critical extension and revision of the manuscript. All authors have read and agreed to the published version of the manuscript.

## Conflicts of Interest

The authors declare no conflicts of interest.

## Data Availability

The authors have nothing to report.
